# Book Reviews

**Published:** 1898-08

**Authors:** 


### *BOOK REVIEWS.*

THE TREATMENT OF CHOLERAIC DIARRHEA. Published by the Lambert Pharmacal Co., St. Louis.

This is a small, neatly bound in cloth book, giving reports of cases and their treatment with drugs put out by the publishers. Though more expensive than the average, the book will be sent gratis to physicians who request it.

REPORT OF THE COMMISSIONER OF EDUCATION FOR THE YEAR 1896–7. Vol. I.

We have just received the first volume of this work from Washington. Judging by this volume, the work taken altogether will be very valuable. It contains matter concerning education, both here and abroad, which will make it an invaluable reference book.

A COMPEND OF DISEASES OF THE SKIN. By Jay F. Schamberg, A.B., M.D. Associate in Skin Diseases, Philadelphia Polyclinic; Dermatologist to the Union Mission Hospital, etc. 99 illustrations. Philadelphia, P. Blakiston's Son & Co. 1898. Price, 80c. net.

This is a very small and concise book. Suitable for quick reference or review study. The best authorities and their illustrations have been liberally used in the preparation of the book. It will suit for the purpose intended. M. B. H.

ATLAS AND ABSTRACT OF THE DISEASES OF THE LARYNX. By Dr. L. Grünwald, of Munich. Authorized Translation from the German. Published by W. B. Saunders. Philadelphia. Price, $2.50.

This volume is one of the series of Atlases which are now being published by this well-known house. Like all of the others, it is excellent. The main feature of the Atlas is, of course, the colored plates representing the various diseases of the larynx. We must say that these are beautifully executed, and will give one a most excellent idea of the disease which the plate represents. Accompanying each plate is the history of the case which it represents, which therefore is doubly interesting. The laryngologist will find this volume a most excellent addition to his library. The publishers are certainly to be congratulated.

---

CONSERVATIVE GYNECOLOGY AND ELECTRO-THERAPEUTICS. A Practical Treatise on the Diseases of Women and their Treatment by Electricity. Third edition. Revised, rewritten and greatly enlarged. By G. Betton Massey, M.D., Physician to the Gynecic Department of Howard Hospital, Philadelphia; Late Electro-Therapeutist to the Infirmary for Nervous Diseases, Philadelphia; Fellow and ex-President of the American Electro-Therapeutic Association, of the Société Française d'Électrothérapie of the American Medical Association, etc. Illustrated with twelve full-page original chromo-lithographic plates in twelve colors, numerous full-page original half-tone plates of photographs taken from nature, and many other engravings in the text. Royal octavo. 400 pages. Extra cloth, beveled edges, $3.50 net. The F. A. Davis Co., Publishers, 1914–16 Cherry St., Philadelphia; 117 W. Forty-second St., New York City; 9 Lakeside Building, 218–220 S. Clark St., Chicago, Ill.

The third edition of this work by Dr. Massey has just been issued from the publishers' press. It shows many changes of value and almost makes a new book. The colored plates showing the different diseases of the cervix uteri are most beautifully executed. The work, of course, treats principally of the electric treatment of the various diseases found in the range of the gynecologist. No one can now doubt the efficacy of this mode of treatment in a large number of cases which consult the gynecologist. Dr. Massey is a pioneer worker, and this volume of his is exceedingly clear and explicit. Being familiar with electric appliances, Dr. M. has made the subject so clear as to be understood by every physician. We predict a rapid demand for this new edition. It is certainly a valuable work.

A SYSTEM OF PRACTICAL MEDICINE. By American Authors. Edited by Alfred Lee Loomis, M.D., Late Professor of Pathology and Practical Medicine in the New York University, and William Gilman Thompson, M.D., Professor of Medicine in the Cornell University Medical College, New York. In four imperial octavo volumes. Volume IV.—Diseases of the Nervous System and Mind; Vasomotor and Trophic Disorders; Diseases of the Muscles; Osteo-Malacia; Rachitis; Rheumatism; Arthritis; Gout; Lithæmia; Obesity; Scurvy; Addison's Disease. 1099 pages, 95 engravings, and 8 full-page plates in colors and monochrome. For sale by subscription. Per volume, cloth, $5.00; leather, $6.00; half-morocco, $7.00. Lea Brothers & Co., Publishers, Philadelphia and New York. 1898.

This, the fourth and last volume of this most excellent work, is before us. The authors have certainly done their work well, and no less can be said of the publishers. It is a monumental work and will long live as such to the name of both its editors. The authors for the various subjects treated in this volume have been well selected, just as they have been for all the previous volumes, and there is no lack of thoroughness in every detail. As a reference book to the System of Medicine it has no superior. It is obliged to meet with success.

---

HAY FEVER AND ITS SUCCESSFUL TREATMENT. By W. C. Hollopeter, A.M., M.D., of Philadelphia. Published by P. Blakiston's Son & Co., Philadelphia. Price, $1.00 net.

This little brochure is just before us. The author has this to say in his preface: "Having had remarkable and uniform success with a simple treatment of hay-fever for the last ten years, during which time I have given complete relief to over two hundred patients in my private practice, and having made a thorough clinical study of this affection, as well as an exhaustive review of the literature relative to it, I feel justified in presenting the results of my labors in this short treatise."

This statement we consider remarkable, for if the treatment has been "uniformly successful," then Dr. Hollopeter has given to the world one of the most remarkable cures in recent years, and a boon to hundreds of sufferers who have looked in vain for this "balm of Gilead."

The author has given us a most excellent review of the history of hay-fever, and also of the literature bearing upon the subject.

The history of hay-fever embraces twelve pages; its exciting causes seventeen pages; its predisposing causes twenty-five pages; and then follows the time of occurrence, duration, symptoms, complications and sequelæ, pathology, diagnosis, prognosis and treatment.

The author says he has been uniformly successful. To sum up in his own words, his treatment consists entirely in this: "By a daily sterilization of the nares and post-nasal spaces, the victims of hay-fever may remain in the city attending to their usual duties surrounded by dust, or in the country amid blooming flowers."

The method of sterilizing these parts is given and the remedies used. It seems strange that so simple a treatment was never discovered before. Experience alone will justify the author's conclusions.

---

### SURGICAL HINTS.

Never allow rubber plaster to come in contact with a surface uncovered by normal skin. Since it cannot be sterilized by heat, it must be considered as dirty.

Before operating, always find out whether the patient has any malarial history. The discovery of this fact will save you many a bad scare when temperature rises suddenly after operation.

As long as any urine issues from the urethra it cannot be said that there is an impassable stricture. Patience and gentleness will do wonders. The most skilful surgeons see very few strictures that prove impassable.

An aseptic dressing placed over a wound that is expected to unite by first intention should be left undisturbed until it is time to remove the stitches, or until there is reason to believe that the case is not running the expected aseptic course.—*International Journal of Surgery.*

